# Stereodynamic Investigation of Labile Stereogenic Centres in Dihydroartemisinin 

**DOI:** 10.3390/molecules15031309

**Published:** 2010-03-05

**Authors:** Ilaria D’Acquarica, Francesco Gasparrini, Dorina Kotoni, Marco Pierini, Claudio Villani, Walter Cabri, Michela Di Mattia, Fabrizio Giorgi

**Affiliations:** 1 Dipartimento di Chimica e Tecnologie del Farmaco, Sapienza Università di Roma, P. le Aldo Moro 5, 00185 Roma, Italy; 2 Analytical Development, R&D Department, sigma-tau S.p.A., Via Pontina km 30,400, 00040 Pomezia, Italy; E-Mail: walter.cabri@sigma-tau.it (W.C.)

**Keywords:** Dihydroartemisinin (DHA), on-column epimerization, cryo-HPLC, dynamic HPLC (DHPLC), computer simulation

## Abstract

Since its identification in the early 1970s, artemisinin, as well as semi-synthetic derivatives and synthetic trioxanes, have been used in malaria therapy. Reduction of artemisinin by NaBH_4_ produced dihydroartemisinin (DHA), and yielded a new stereochemically labile centre at C-10, which, in turn, provided two interconverting lactol hemiacetal epimers (namely α and β), whose rate of interconversion depends on buffer, pH, and solvent polarity. Since interconversion of the two epimers occurred on a chromatographic time-scale, this prompted a thorough investigation of the phenomenon as a crucial requisite of any analytical method aimed at quantitating this family of drugs. In this critical review we discuss the current importance of the on-column epimerization of DHA in the development of analytical methods aimed at quantifying the drug, with the purpose of identifying the optimal conditions to minimize on-column epimerization while achieving the best selectivity and efficiency of the overall separation.

## 1. Introduction

**Figure 1:**
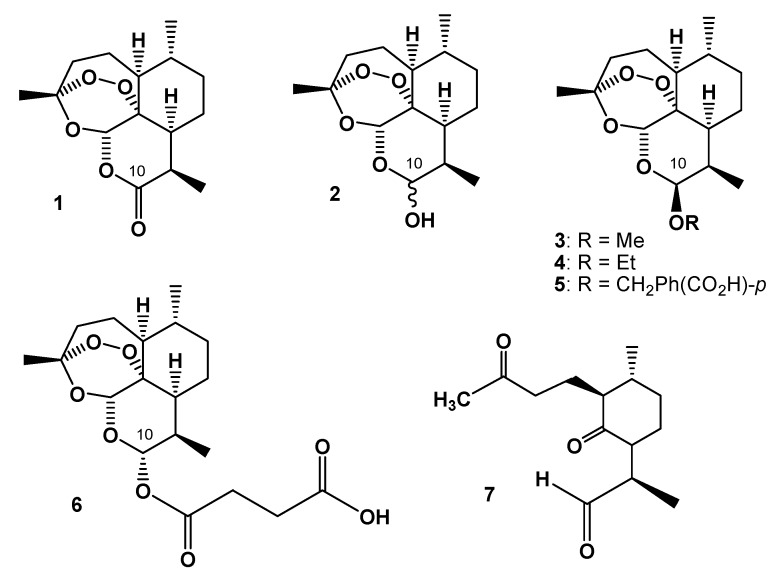
Chemical structures of artemisinin (**1**), dihydroartemisinin (DHA, **2**), artemether (**3**), arteether (**4**), artelinic acid (**5**), artesunic acid (**6**), and a ubiquitous thermal decomposition product of **2** designated as diketoaldehyde (DKA, **7**).

Artemisinin (Qinghaosu, **1**, Figure 1) is a sesquiterpene lactone endoperoxide isolated from *Artemisia annua* L. that Chinese herbalists have traditionally used to treat malaria [1,2], a dramatic cause of death and illness in children and adults in tropical countries. The combination of artemisinin derivatives with other effective antimalarial medicines (artemisinin-based combination therapies or ACTs) is currently the most effective treatment for *Plasmodium falciparum* malaria – the most lethal form of the disease [3]. Since its identification in the early 1970s, artemisinin as well as semi-synthetic derivatives [4] and synthetic trioxanes [5] have been utilized in therapy. Reduction of artemisinin by sodium borohydride in methanol [6] produces dihydroartemisinin (DHA, **2**, Figure 1), which is also its main metabolite and provides improved antimalarial potency [5]. The synthesis of **2** opened pathways for further derivatization at C-10 to give ether (**3**-**5** in Figure 1) and ester (**6** in Figure 1) derivatives, largely exploited by the China Cooperative Research Group [7] with the aim of tuning water and/or oil solubility and improving bioavailability.

**Figure 2:**
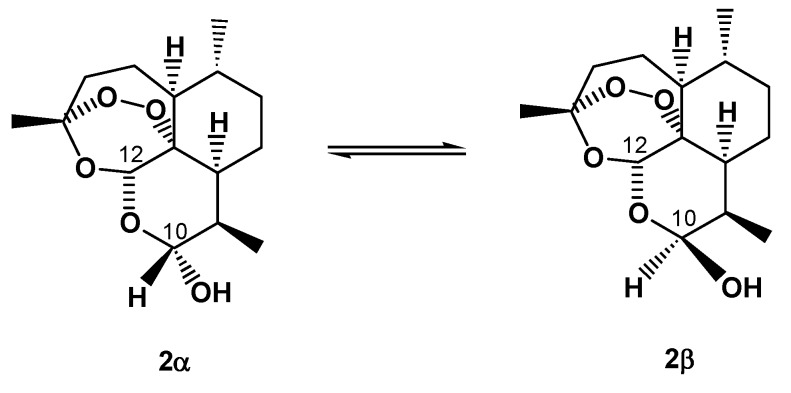
Chemical structures of the two interconverting epimers of DHA (**2**): the 2α-epimer bears the hydroxyl group in the equatorial position (absolute stereochemistry at C-10: *R*), whereas the 2β-epimer possesses an axial hydroxyl group.

**Figure 3:**
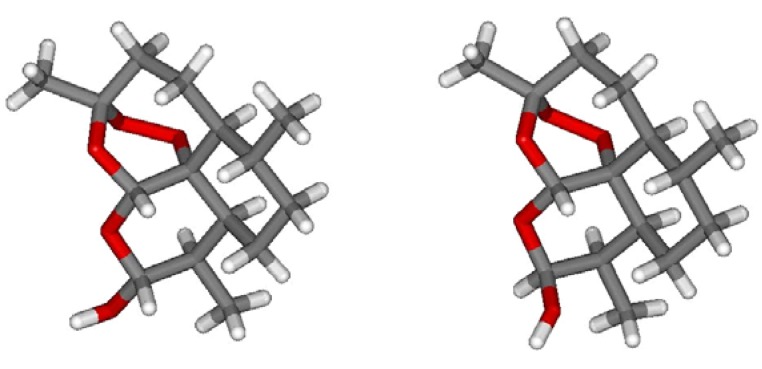
Polytube models of the two interconverting epimers of DHA (**2**): the 2β-epimer (right) was obtained by computer editing of the X-Ray data of crystalline **2β** [10]; the model for the 2α-epimer (left) was derived by molecular mechanics optimization (MMFF force field as implemented in SPARTAN 04) by inverting the configuration at C-10.

Conversion of the lactone carbonyl group at C-10 of artemisinin into the hydroxyl (hemiacetal) group in **2** yielded a new stereochemically labile centre in the molecule, which, in turn, provided two interconverting lactol hemiacetal epimers, namely **2α** and **2β** (Figure 2). The 2α-epimer bears the hydroxyl group in the equatorial position (absolute stereochemistry at C-10: *R*), whereas the 2β-epimer possesses an axial hydroxyl group [8], as shown by polytube models in Figure 3.

Notably, although **2** has a chair-like pyranose ring, such nomenclature is the reverse of that normally used for designating the stereochemistry of sugars and glycosides, in which, for example, the α-D-glucopyranose possesses an axial hydroxyl group [9].

Bulk solid **2** consists exclusively of the β-epimer, as illustrated by an X-ray crystallographic study on crystalline **2** [10], however, upon dissolution in various solvents, the initial solutions consisting of **2β** slowly equilibrate to mixtures of **2α** and **2β** epimers with different solvent-dependent compositions [9,10,11,12]. The **2α** epimer has not been isolated in the solid state. Computational studies, performed with AM1 and PM3 semiempirical methods, showed that the DHA epimers have very close heats of formation, *i.e.*, have similar thermodynamic stabilities [11]. It has also been found that acylations where the hydroxyl group of **2** acts as the nucleophile exclusively yield the α-acyl derivatives for kinetic reasons. The biological implication of such a process is the observation that phase II glucuronidation of **2** exclusively provides the α-DHA-β-glucuronide [13,14]. Thus, a deeper understanding of the kinetic, thermodynamic and mechanistic features of the **2α/2β** equilibration (*i.e.*, epimerization) may have a great importance in the investigation of the mechanism of action and/or toxicity of the drug at molecular level. Moreover, since a previous study [15] on the equilibrium between **2α** and **2β** showed that interconversion of the two epimers occurred on a chromatographic time-scale, this prompted a thorough investigation of the phenomenon as a crucial requisite of any analytical method aimed at quantifying this family of drugs.

## 2. On-Column Interconversions

Investigations of dynamic molecular processes and determination of their kinetic parameters are commonly performed by dynamic nuclear magnetic resonance (DNMR) spectroscopy [16,17,18,19], which yields quite reliable values for the corresponding free energies of activation (Δ*G*^#^). Pertinent kinetic parameters might also be determined by chromatography, specifically for two species that interconvert during their passage through the column itself (*i.e.*, on-column interconverting species).

**Scheme 1:**
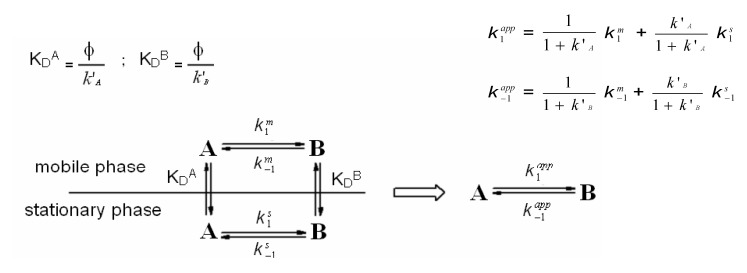
Primary (vertical arrows) and secondary (horizontal arrows) equilibria taking place during chromatography of two interconvertible species A and B on a stationary phase. Left: actual interconversions occurring in the mobile and stationary phases; right: the apparent equilibria. *k*’_A_ and *k*’_B_ are the retention factors of the first (A) and second (B) eluting species, *k*_1_^m^ and *k*_−1_^m^ are the rate constants for the forward and backward interconversion in mobile phase, respectively, and *k*_1_^s^ and *k*_−1_^s^ are the rate constants for the forward and backward interconversion in stationary phase, respectively.

Chromatographic retention of a species, in its simplest form, is related to the equilibrium constant for its distribution between the mobile and stationary phases (Scheme 1). Chromatography of two interconverting species, however, leads to the establishment of a secondary equilibrium. When an analyte is subjected to a secondary equilibrium, its retention is a weighted average of the retention of the two species [20].

**Figure 4:**
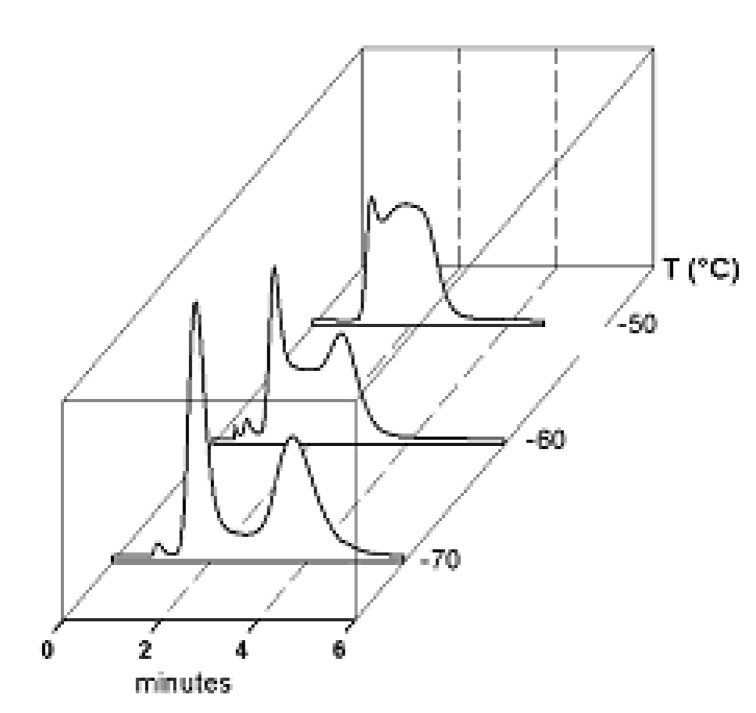
DHPLC traces of a tertiary amide in the form of variable temperature HPLC.

If the interconversion rate is slow compared to the chromatographic process, two resolved peaks are observed due to the occurrence of little or no interconversion. If the rate of interconversion is fast compared to the chromatographic process, only one peak is observed due to the extensive interconversion. However, if the interconversion is on a time-scale similar to that of the chromatographic process, band spreading and peak distortion may be observed (Figure 4). The two peaks may be joined by an elevated baseline (plateau). This elevated baseline represents species which have undergone at least one interconversion cycle during elution through the column.

Stereolabile species can therefore be conveniently investigated by Dynamic High Performance Liquid Chromatography (DHPLC), either in the form of variable temperature or variable flow chromatography. A quite comprehensive view of milestone works on dynamic chromatography and its applications is given in refs [21,22,23,24,25,26,27,28,29,30,31,32,33,34,35], including gas and liquid chromatography. DHPLC has been recently used for the study of internal molecular dynamics of a range of chiral stereolabile compounds, and for the determination of kinetic parameters for the pertinent equilibrium (*i.e.*, the reversible isomerization of one enantiomer into the other, or enantiomerization).

**Figure 5:**
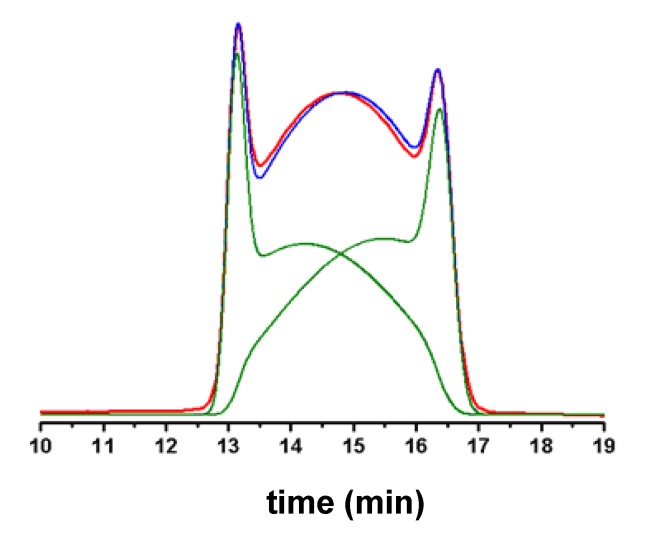
Computer-simulation of experimental deformed profiles of two interconverting species. Red line: experimental profile. Blue line: computer-simulated profile. Green lines: computer-simulated profiles of the individual interconverting species.

In a general DHPLC experiment, the chromatographic column acts at once as chemical reactor and separation device: first-order, reversible processes where the interconversion rates are on the same time-scale as the column separation rate typically yield temperature- and flow-dependent chromatographic profiles, with an interference regime between the two resolved peaks (Figure 5). Computer simulation of the experimental deformed profiles can be used to obtain overall rate constants for the interconversion process occurring during HPLC (Figure 5).

These rate constants are averaged values that bring contributions from the process occurring in the mobile phase (*k*_1_^m^ and *k*_−1_^m^, for the forward and backward interconversion, respectively) and in the stationary phase (*k*_1_^s^ and *k*_−1_^s^, for the forward and backward interconversion, respectively). If one of the two constants (usually *k*^m^) is available from independent measurements, the missing rate constant can be obtained by simulation [27].

Several methods have been used to extract kinetic data from experimental elution profiles (the theoretical plate model [27], the stochastic model [26,33], the continuous flow model [25], peak deconvolution methods [32,33], approximation functions [31], and the unified equation [35]), and each method exploits a different theoretical framework to describe the dynamic system in which primary and secondary equilibria take place inside the column (Scheme 1). The theoretical plate model portrays the chromatographic separation as a discontinuous process, by assuming that all the steps continually proceed in separate uniform sections of a column consisting of N plates. Each theoretical plate is then considered as a distinct chemical reactor in which three events take place: distribution of the species between the mobile and stationary phases, interconversion in both phases, and shifting of the mobile phase onto the next plate [27]. The stochastic model describes the chromatographic separation using time-dependent distribution functions [26,33]. The elution profile of two interconverting species is given by the sum of the distribution functions of the non-interconverted species and the probability of density functions of the interconverted species. In the continuous flow model, general analytical expressions based on the dimensionless Damköhler (Da) number describe the chromatographic mass distribution at the column outlet for a solute subjected to secondary equilibria [25]. In contrast to these ab initio-type simulations, semiempirical peak deconvolution methods have also been developed to estimate rate constants of enantiomerization and isomerization [32,33]. A direct calculation of enantiomerization barriers from chromatographic parameters based on an approximation function was also attempted [31], limited to equilibrated and degenerated first-order reactions, *i.e.*, enantiomerizations. Later on, a unified equation has been derived [35] to calculate rate constants of reversible as well as irreversible (pseudo-) first-order reactions by a few iterative steps, without the need of performing a computationally extensive simulation of elution profiles. This calculation method is a valuable tool for the investigation of first-order reactions, in particular the stereochemical integrity of enantiomeric, epimeric, and isomeric compounds. In this context, it is worthy of noting that we have developed a lab-made computer program, Auto DHPLC y2k, which implements both stochastic [26,33] and theoretical plate [27] models, and may take into account all types of first-order interconversions, *i.e. ,* enantiomerizations as well as diastereomerizations or constitutional isomerizations (e.g. pseudo first-order tautomerizations), as well as tailing effects [36,37,38,39,40,41]. Within non-enantiomeric isomerizations, forward and backward processes occur at different rates in the achiral mobile phase, where the two isomerizing species are usually present in differing amounts. According to the thermodynamic cycle involved inside a virtual chromatographic theoretical plate for a generic first-order isomerization process concomitant with the chromatographic distribution equilibria (see Scheme 1), we applied in the algorithm the following general equation:

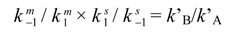
(1)
where *k*’_A_ and *k*’_B_ are the retention factors of the first (A) and second (B) eluting species, *k*_−1_^m^ and *k*_1_^m^ are the rate constants for the backward and forward interconversion in mobile phase, respectively, and *k*_1_^s^ and *k*_−1_^s^ are the rate constants for the forward and backward interconversion in stationary phase, respectively. Program functionality was validated on several first-order isomerizations (both enantiomerization and non-enantiomerization) by comparing DHPLC results with those obtained by DNMR technique [36,37,38,39] or by classical method [40]. The algorithm also implements the chance of taking tailing effects into account. Both chromatographic and kinetic parameters can be automatically optimized by simplex algorithm until obtaining the best agreement between experimental and simulated dynamic chromatograms.

## 3. HPLC Analytical Methods for DHA

Numerous HPLC methods [42,43,44,45,46,47,48,49,50,51,52,53,54,55,56,57] have been developed for the analysis and monitoring of plasma levels of **2**, typically based on two main detection strategies due to the lack of strong ultraviolet absorption or fluorescent chromophores: reductive electrochemical (EC) [42,45,46,47,56] and UV detection [43,44,53], with pre- or post-column derivatization. The latter approach lacks specificity in that metabolites of the drug are also converted, in many instances, to products having overlapping UV spectra. On the other hand, HPLC-EC provides excellent specificity and sensitivity, although it suffers from some inherent difficulties, *i.e.*, rigorous deoxygenation of samples and mobile phases, and special laboratory facilities are needed. To overcome these shortcomings, evaporative light scattering detection (ELSD) was also coupled to the HPLC analysis of artemisinin and related analogues [48]. The high sensitivity and selectivity of mass spectrometry (MS) opened the way to a proliferation of analytical methods based on HPLC-MS coupling [49,50,51,52,55,57] for the plasma monitoring of **2**, either based on atmospheric pressure chemical ionization (APCI) [50,55] or electrospray ionization (ESI) mode [49,51,52,57]. Radiochromatographic detection [54] was exploited as well in a recent HPLC study aimed at determining the **2α**/**2β** ratios *in vivo* and evaluating the protein binding of **2**.

Notwithstanding such a wide availability of literature on robust HPLC methods suitable for the analysis of **2**, none of them considers the effects of epimer equilibration on the analytical response. Actually, the very first study showing that interconversion of the two DHA epimers (Figure 2) occurred on a chromatographic time-scale (*i.e.*, on-column epimerization could be detected) was performed in 1986 by investigating the multiple-peaks of the drug by reversed-phase HPLC [15], but no other related papers appeared in literature afterwards. Moreover, the author of that study reported a greatly overestimated activation energy barrier of 280 kJ mol^−^^1^ for the interconversion of the two DHA epimers, which cannot be related to dynamic events occurring on the chromatographic time-scale at room temperature [58]. Only recently [41], was this issue considered with the aim of providing improved and reliable HPLC procedures suited for either separation of **2α** and **2β** or their quantification in solution and in complex mixtures, under conditions of suppressed interconversion. In such study, we developed an effective analytical approach to overcome the intrinsic shortcomings of the International Pharmacopoeia guidelines on antimalarial drugs.

## 4. Pharmacopoeia Guidelines on Antimalarial drugs

In the International Pharmacopoeia monograph on Artenimolum or Artenimol (*i.e.*, dihydroartemisinin) [59], the method currently recommended is an HPLC assay based on the use of a stainless steel column (100 mm × 4.6 mm I.D.) packed with a reversed-phase (RP) C_18_ stationary phase (3.0 μm particle size). The mobile phase is acetonitrile-water 60:40 (v/v), delivered at a flow-rate of 0.6 mL min^−^^1^ and the detection system used is an ultraviolet spectrophotometer set at a wavelength of about 216 nm. The aim of the pharmacopoeial method is to quantitate DHA (**2**) in the presence of artemisinin (**1**) as related substance. The monograph does not provide for any other related substance. The final requirement is that “*the test is not valid unless the relative retention of* *α-artenimol compared with artemisinin is about 0.6, and the resolution between the peaks is not less than 2.0. […] Measure the areas of the peak (twin-peak) responses and calculate the percentage content of C_15_H_22_O_5_ (i.e., artemisinin) with reference to the dried substance*”. Four main items are raised when considering the above method: (*i*) the column temperature is not specified; (*ii*) no mention is made of the interconversion between the two epimers of **2**, which indeed occurs on the chromatographic time-scale; (*iii*) the conditions are not very selective towards DKA (**7**, Figure 1), which is a ubiquitous contaminant of **2**, and (*iv*), since signal-to-noise ratios in the presence of plateau zones are always smaller than in normal elution profiles, quantitation of species eventually eluting in the plateau area would be negatively affected. The main drawback of the method, however, is that the presence of a visible plateau between the two DHA epimers (they called it twin-peak) is completely neglected. In addition, stationary phases may have a retarding or activating effect on the kinetics of the dynamic process involving stereolabile species [20], and this feature should be taken into account as well.

**Figure 6:**
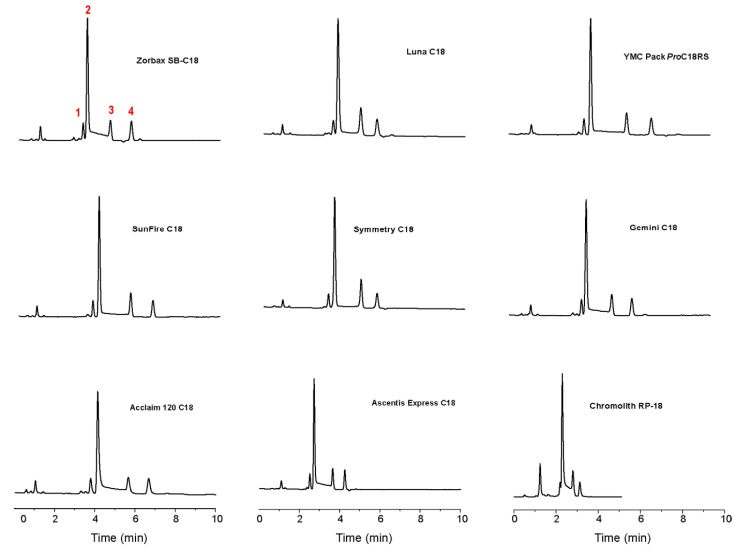
Typical room temperature chromatograms obtained for a standard mixture of **1** and **2** (containing **7** as impurity). Peak 1 corresponds to **7**, peak 2 to the 2α-epimer, peak 3 to the 2β-epimer, and peak 4 to **1**. Reproduced from Figure 3 in Reference [41] with permission from Elsevier. Copyright 2008.

## 5. On-Column Epimerization of DHA

To overcome the intrinsic shortcomings of the International Pharmacopoeia and try to address the four points raised when considering such method, we have performed a systematic investigation of the influence of chromatographic conditions (stationary phase and column temperature) on the simultaneous reversed-phase HPLC determination of artemisinin (**1**), α-DHA (**2α**), β-DHA (**2β**), and a thermal decomposition product of **2** (DKA, **7**), taking into consideration for the first time the on-column epimerization of **2** [41]. Nine commercial RP-C_18_ columns with different pore size, surface area, carbon load and permeability were evaluated according to the International Pharmacopoeia monograph on DHA (see Figure 6), and compared on the basis of their stationary phase effect towards the interconversion between **2α** and **2β**. In particular, we defined the Catalytic Effect of Stationary Phases (CESP) as the percentage increasing (positive) or decreasing (negative) of the epimerization rate constant, with respect to the analogous value measured in mobile phase.

**Figure 7:**
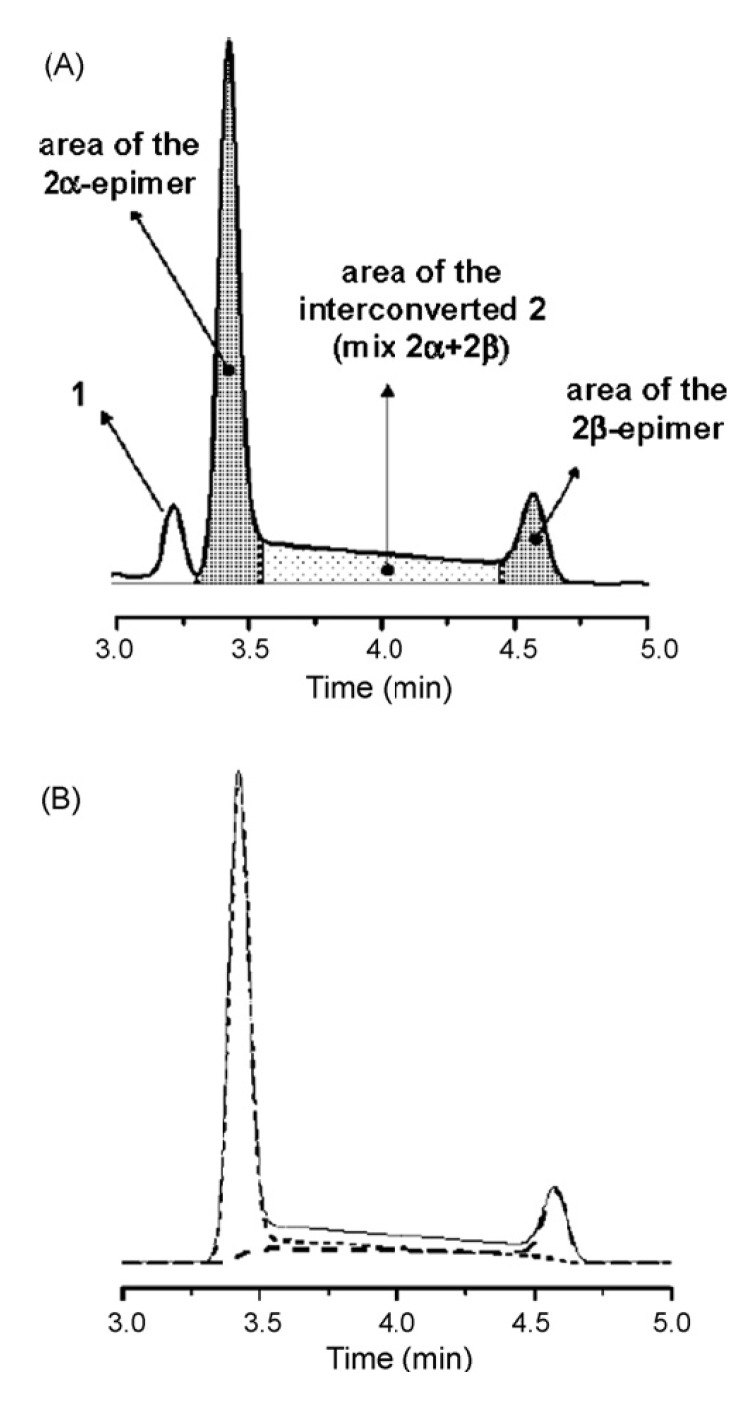
(A) Schematic representation of the integration mode used for the calculation of 2α- and 2β-epimers areas. (B) Computer simulated profiles of **2α** (dotted line), **2β** (dashed line), and of the mixture **2α**+ **2β** (solid line). Reproduced from Figure 5 in Reference [41] with permission from Elsevier. Copyright 2008.

Activation free energies of the process, obtained by computer simulation of the experimentally obtained elution profiles (see Figure 7), showed that the Symmetry C_18_ column among the nine analyzed has an inhibiting effect on epimerization (Δ*G*^#^ = 22.1 for **2α**→ **2β** and 21.6 kcal mol^−1^ for the backward process, compared to Δ*G*^#^ = 21.7 and 21.0 kcal mol^−^^1^ for the same processes in free solution).

This column was therefore selected for the development of a cryo-HPLC method aimed at minimizing on-column interconversion. Variable temperature (from 40 to 0 °C) separations performed on the Symmetry C_18_ column allowed to detect plateau zones only at *T* > 20 ° C. For this reason, only simulations of the chromatograms registered from 40 to 25 °C were made, by using the classical stochastic model, as implemented within the Auto DHPLC y2k program [36,37,38,39,40]. Since the **2α**/**2β** ratio was shown to be temperature-independent within the explored range, a constant value of the relative abundances of the two epimers was set in all the performed relevant simulations.

**Figure 8:**
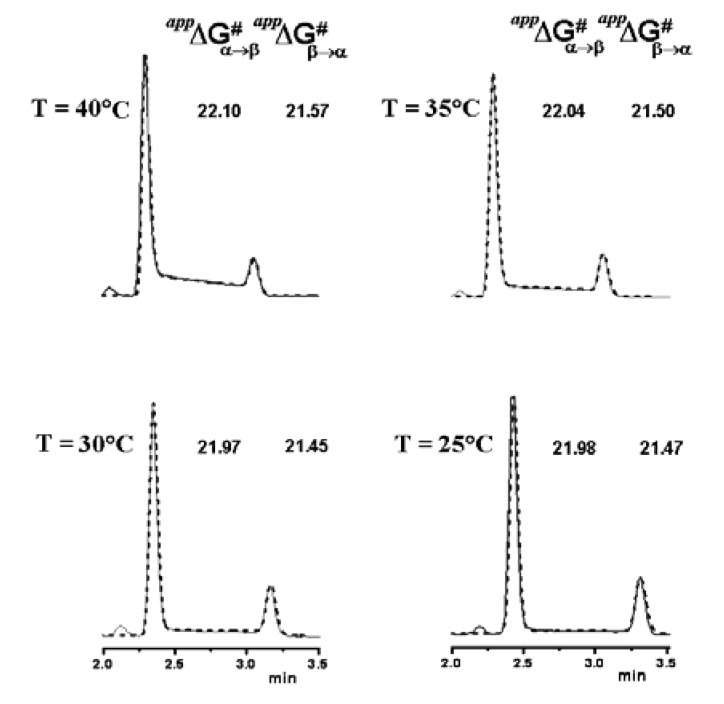
Variable temperature chromatographic profiles obtained on the Symmetry C_18_ column. Solid line: experimental chromatograms. Dotted line: computer simulated profiles obtained with the measured free energy activation barriers for the on-column epimerization process. Reproduced from Figure 7 in Reference [41] with permission from Elsevier. Copyright 2008.

On the basis of the epimerization rate constants extrapolated at *T* < 25 °C, we calculated a marginal plateau area (< 2%) only close to *T* = 0 °C for the Symmetry C_18_ column, clearly showing that low column temperatures are necessary to suppress interconversion. Figure 8 shows the superimposed experimental and simulated chromatographic profiles with the measured activation free energies. Van’t Hoff analyses of the obtained data were also carried out to evaluate the enthalpic and entropic contributions to the epimerization barrier. We found that just a quite small contribution to Δ*G*^#^_α__–__β_ and Δ*G*^#^_β__–__α_ is due to entropy (Δ*S*^#^ values < 10 u.e.). This suggested that a monomolecular process may be involved in the rate-determining step, reasonably, the ring opening of either the protonated or deprotonated α- or β-hemiacetalic form of **2**, generated in a previous reversible step by reaction with an acid or a base, respectively. Such kinetic pathway would be in agreement to what already reported [12].

In conclusion, a full thermodynamic and kinetic investigation of the DHA interconversion [58] will shed light on the effective ratio of the two epimers *in vivo*, irrespective of the isomeric purity at which the drug would have been administered, as well as on their preferential protein binding. Moreover, since artemisinin and its synthetic derivatives are discovered to be additionally endowed with anticancer, antiangiogenesis, antiviral, immunosuppressive, and antifungal activity, they also represent a very promising field in drug discovery [60,61].

## 6. Conclusions

Artemisinin derivatives such as DHA, artesunate, and artemether are nowadays playing an increasing role in the treatment of drug-resistant malaria. They are the most potent antimalarials available, rapidly lethal to all asexual stages of the parasite *Plasmodium falciparum*. DHA is the main metabolite of artemisinin, and contains a stereochemically labile centre at C-10, which, in turn, provides two interconverting lactol hemiacetal epimers, namely α and β. DHA at the solid state consists exclusively of the β-epimer, however, upon dissolution in various solvents, the initial solutions slowly equilibrate to mixtures of α- and β-epimers with different solvent-dependent composition. Thus, a deeper understanding of the kinetic, thermodynamic and mechanistic features of the DHA equilibration (*i.e.*, epimerization) may prove of great importance in the investigation of the mechanism of action and/or toxicity of the drug at molecular level. Moreover, since the equilibrium between the two DHA epimers occurs in a chromatographic time-scale, a crucial requisite of any chromatographic method aimed at quantitating this family of drugs is the identification of some optimal conditions (such as stationary phase and column temperature) able to suppress or at least minimize on-column epimerization, while achieving the best selectivity and efficiency of separation. Actually, in the International Pharmacopoeia monograph on DHA, the drug is quantitated in the presence of artemisinin as related substance. No other related substance is contemplated. The main drawback of the method, however, is that the presence of a visible plateau between the two interconverting epimers is completely neglected. Despite the large availability in literature of robust HPLC methods suitable for the analysis of DHA, none of them considers the effects of epimers equilibration on the analytical response. Only recently, this issue was considered with the aim of providing improved and reliable HPLC procedures suited for either separation of **2α** and **2β** or their quantification in solution and in complex mixtures. Dynamic High Performance Liquid Chromatography (DHPLC) and computer simulation of the experimental deformed profiles clearly showed that low column temperatures are necessary to suppress interconversion between DHA epimers.
